# Antimicrobial resistance in *Neisseria gonorrhoeae* and its risk groups in 23 European countries in 2022 within the European Gonococcal Antimicrobial Surveillance Programme (Euro-GASP): a retrospective observational study

**DOI:** 10.1016/j.lanepe.2025.101318

**Published:** 2025-05-10

**Authors:** Susanne Jacobsson, Michelle J. Cole, Daniel Schröder, Melissa Jansen van Rensburg, Michaela Day, Csaba Ködmön, Magnus Unemo, Euro-GASP study group, Sonja Pleininger, Stefanie Schindler, Ziad El-Khatib, Irith De Baetselier, Dorien Van den Bossche, Dominique Van Beckhoven, Amaryl Lecompte, Ivva Philipova, Hana Zákoucká, Helena Žemličková, Vladislav Jakubů, Steen Hoffmann, Maria Wessman, Kairi Tõnsau, Suvi Korhonen, Jukka Torvikoski, Jari Jalava, Beatrice Bercot, Emilie Chazelle, Cheick Kounta, Gilles Delmas, Dagmar Heuer, Regina Selb, Klaus Jansen, Vivi Miriagou, Vasilios Raftopoulos, Dimitra Paraskeva, Eszter Balla, Lena Rós Ásmundsdóttir, Kristjan Orri Helgason, Anna Margret Gudmundsdottir, Marianna Thordardottir, Brendan Crowley, Sinead Saab, Candice Principe, Derval Igoe, Mark Campbell, Angeline McIntyre, Paola Stefanelli, Barbara Suligoi, Francesca Vella, Robert Cassar, Julie Haider, Alje Van Dam, Maartje Visser, Dominique Caugant, Hilde Kløvstad, Beata Młynarczyk-Bonikowska, Maria José Borrego, Peter Pavlik, Alexandra Bražinová, Tanja Kustec, Irena Klavs, Polona Maver Vodičar, Raquel Abad Torreblanca, Julio Vazquez Moreno, Javier Gómez Castellá, Magnus Unemo, Susanne Jacobsson, Daniel Golparian, Daniel Schröder

**Affiliations:** a WHO Collaborating Centre for Gonorrhoea and Other STIs, National Reference Laboratory for STIs, Department of Laboratory Medicine, Faculty of Medicine and Health, Örebro University, Örebro, Sweden; b UK Health Security Agency (UKHSA), London, UK; c European Centre for Disease Prevention and Control (ECDC), Stockholm, Sweden; d Institute for Global Health, University College London (UCL), London, UK

**Keywords:** Gonorrhoea, *Neisseria gonorrhoeae*, Treatment, Antimicrobial resistance, Surveillance, ECDC, Europe

## Abstract

**Background:**

Since 2009, the European Centre for Disease Prevention and Control (ECDC) has coordinated the European Gonococcal Antimicrobial Surveillance Programme (Euro-GASP) to monitor antimicrobial resistance (AMR) in *Neisseria gonorrhoeae* across the European Union and European Economic Area (EU/EEA). The aims of this study were to report Euro-GASP 2022 data and to compare with the most recently published Euro-GASP data (from 2016 to 2019), to identify changes in AMR and in risk groups for AMR.

**Methods:**

In this observational study, 23 EU/EEA countries submitted AMR data for gonococcal isolates from 2022, linked to patient epidemiological data, to The European Surveillance System (TESSy). Statistical analyses (Z-test) were used to determine the significance of the differences between the epidemiological data and proportion of AMR isolates in 2022 versus 2019 and 2016. The risk factors associated with AMR isolates were assessed using univariate and multivariable logistic regression analyses of odds ratios.

**Findings:**

Ceftriaxone resistance in 2022 (0.03%, 1/3008) remained low (0.06% (2/3239) in 2019), and cefixime resistance (0.3%, 10/3008) had decreased (0.8% (26/3239) in 2019). Azithromycin resistance (24.9%, 749/3008) and ciprofloxacin resistance (65.8%, 1980/3008) had increased (9.0% (284/3159) and 57.4% (1665/2884), respectively, in 2019). A marked increase in the number (575; 502 in 2019) and proportion (19.2%; 15.8% in 2019) of female gonorrhoea cases was also identified in 2022. In the univariate analysis, azithromycin resistance was associated with oropharyngeal (OR 1.67, CI 1.28–2.18; p < 0.0001) and anorectal infections (OR 1.38, CI 1.08–1.76; p = 0.0094), men-who-have-sex-with-men (MSM) (OR 3.88, CI 2.80–5.37; p < 0.0001), and females (1.71, CI 1.21–2.41; p = 0.0022). In the multivariable logistic regression model, only azithromycin resistance and MSM remained associated (OR 2.85, CI 1.33–4.73; p = 0.0040).

**Interpretation:**

While ceftriaxone resistance remains sporadically detected in Euro-GASP, the increase in reports of occasional ceftriaxone resistance in EU/EEA countries and substantial increase in azithromycin resistance underscore the urgent need for enhanced AMR surveillance. The Euro-GASP data is crucial for refining treatment guidelines and mitigating the spread of AMR gonococcal strains. Novel effective antimicrobials for gonorrhoea treatment remain imperative.

**Funding:**

10.13039/501100000805ECDC.

**Research in context**

**Evidence before this study**

Gonorrhoea is a public health concern in Europe, where the incidence is increasing, and globally. Antimicrobial resistance (AMR) in *Neisseria gonorrhoeae* is compromising the management and control of gonorrhoea, and recent reports from several countries have described resistance to the last option for empiric first-line treatment, that is, ceftriaxone. Surveillance of gonococcal AMR has been stated essential by the WHO, ECDC and many national public health organisations. We searched PubMed for publications in English language using the terms “*N. gonorrhoeae*” AND “surveillance” AND “Europe” AND “antimicrobial susceptibility” OR “antimicrobial resistance” up to Jan 1, 2025. The most recent published collated European *in-vitro* AMR data were from 2019, and the main conclusions of this paper included that ‘it is imperative that the MICs for *N. gonorrhoeae* isolates continue to be monitored very closely over the next years, particularly of azithromycin and ceftriaxone.’ The paper describing European gonococcal AMR data from 2019 as well as a similar paper describing European data from 2016 were used for comparison to our presently published European gonococcal AMR data from 2022.

**Added value of this study**

We report gonococcal AMR data from 2022 for 23 European Union and European Economic Area (EU/EEA) countries. In total, 3008 *N. gonorrhoeae* isolates, linked to patient epidemiological data, were examined for their susceptibility to ceftriaxone, cefixime, azithromycin, and ciprofloxacin. In 2022, resistance to ceftriaxone and cefixime remained low, at 0.03% and 0.3%, respectively. However, resistance to azithromycin and ciprofloxacin had increased to 24.9% and 65.8%, respectively. In univariate analysis, azithromycin resistance was associated with oropharyngeal and anorectal infections, men-who-have-sex-with-men (MSM), and females. In the multivariable logistic regression model, azithromycin resistance remained associated with MSM. Notably, the number and proportion of female gonorrhoea cases had significantly increased in 2022.

**Implications of all the available evidence**

Gonorrhoea could become untreatable due to high AMR levels worldwide. Resistance to the last remaining option for empiric first-line treatment of gonorrhoea, ceftriaxone, is already high in several Asian countries, and ceftriaxone-resistant gonococcal strains are imported into Europe and other settings. This necessitates continued quality-assured gonococcal AMR monitoring. However, in EU/EEA the susceptibility to ceftriaxone and cefixime remains high, although the MIC values for both these extended-spectrum cephalosporins and the number of occasional reports of ceftriaxone-resistant and cefixime-resistant isolates in EU/EEA countries appear to be increasing, which warrant further monitoring. Furthermore, the rapidly increasing azithromycin resistance observed in 2022 is a major concern and emphasises the importance of a review of the 2020 European gonorrhoea management guideline. The number and proportion of gonorrhoea cases among young females also significantly increased, which underscores the need for continued surveillance and response efforts. Finally, a Euro-GASP 2023 WGS study has been recently initiated. This study will address the fluctuations in gonococcal AMR and the changing gonorrhoea epidemiology in EU/EEA.

## Introduction

The European Gonococcal Antimicrobial Surveillance Programme (Euro-GASP), co-ordinated by the European Centre for Disease Prevention and Control (ECDC), has since 2009 monitored and strengthened the surveillance of antimicrobial resistance (AMR) in *N. gonorrhoeae* in the European Union and European Economic Area (EU/EEA). Euro-GASP activities include centralised and decentralised antimicrobial susceptibility testing (AST) of *N. gonorrhoeae* isolates, epidemiological reporting of Euro-GASP data,^1^ external quality assessment (EQA) schemes,^2^ whole genome sequencing (WGS) of *N. gonorrhoeae*,^3^ laboratory capacity assessment across the EU/EEA, and training in sexually transmitted infection (STI) diagnostics and WGS. The quality-assured data produced are used to detect emerging gonococcal AMR, monitor trends in AMR and to inform international and national treatment guidelines.

Gonorrhoea is the second most frequently notified STI in the EU/EEA, i.e., after chlamydia. After a continuous rise from 2008 to 2019, rates of gonorrhoea abruptly decreased in 2020, i.e., during the first year of the COVID-19 pandemic with its national lockdowns and social restrictions.^4^ Changes in healthcare-seeking behaviour, disruptions in sexual health services, and reduced testing volumes contributed to this decrease. Additionally, underreporting, as resources were redirected toward the COVID-19 response, was a significant contributing factor.^5^ In 2022, however, the gonorrhoea notification rate in EU/EEA was the highest recorded since the European surveillance of STIs began in 2009, with 70,881 confirmed cases (17.9 cases per 100,000 population).^4^

Apart from rising gonorrhoea notification rates, significant challenges in managing gonorrhoea are the increasing levels of resistance to clinically relevant antimicrobials along with verified treatment failures. *N. gonorrhoeae* has developed resistance to various antibiotics over time, including penicillin, tetracycline, earlier generations of macrolides and cephalosporins, and fluoroquinolones.^1^^,^^3^^,^^4^^,^^6^^,^^7^ Currently, the only remaining effective option for empirical first-line monotherapy is the third-generation, extended-spectrum cephalosporin ceftriaxone.^6^ The 2020 European management guideline for gonorrhoea recommends treatment regimens for urogenital and anorectal gonorrhoea consisting of i) ceftriaxone 1 g (intramuscularly) combined with azithromycin 2 g (orally) or ii) ceftriaxone 1 g monotherapy, i.e., in countries with robust AMR surveillance indicating no resistance to ceftriaxone, and where test-of-cure is implemented.^7^ The latter regimen should be supplemented with 100 mg of doxycycline taken twice daily for 7 days if concomitant chlamydia infection has not been excluded with molecular diagnostics. If the first-line treatment fails in treating gonorrhoea, there is currently no ideal second-line treatment. However, several alternative treatments have been suggested, including spectinomycin 2 g together with azithromycin 2 g, gentamicin 240 mg in combination with azithromycin 2 g, or ertapenem 1 g once daily for 3 days.^6^^,^^7^ The most recent Euro-GASP data published in the scientific literature were from 2019,^1^ and more recent data to inform the European management guideline for gonorrhoea^7^ are imperative.

The objectives of the present study were to: i) report Euro-GASP 2022 data, including to combine gonococcal AMR data with demographic, epidemiological and clinical data of the gonorrhoea patients in 23 European countries, with special emphasis on identifying associations between AMR and the patients’ data, and ii) compare the Euro-GASP 2022 data with particularly the published Euro-GASP 2016 data^8^^,^^9^ and Euro-GASP 2019 data,^1^ to identify changes in AMR and in risk groups for AMR. These data are used to monitor and identify trends in the AMR in *N. gonorrhoeae* and the corresponding gonorrhoea patients in EU/EEA and the data are imperative to inform revisions of the European gonorrhoea management guideline^7^ and the national gonorrhoea treatment guidelines in many EU/EEA countries.

## Methods

This was a retrospective observational study examining Euro-GASP 2022 data from 23 European countries compared to particularly the published Euro-GASP 2016 data^8^^,^^9^ and Euro-GASP 2019 data.^1^

### European Gonococcal Antimicrobial Surveillance Programme (Euro-GASP)

Countries participating in Euro-GASP each year collect consecutive *N. gonorrhoeae* isolates (ideally 100–200 isolates depending on the number of gonorrhoea cases reported annually in the country), as previously described.^1^, ^2^, ^3^ Centralised or decentralised AMR testing are performed and antimicrobial susceptibility profiles and patient epidemiological data including site of specimen collection, sex, age, mode of transmission (‘sexual orientation’), previous gonorrhoea diagnosis, HIV status, country of birth, probable country of infection and treatment used, where available, are collected and uploaded by the nominated contact points in each participating country to The European Surveillance System (TESSy). Notably, no ethnicity data is collected. The patient epidemiological data to collect have been agreed by the ECDC, European STI Network, including representatives of all EU/EEA countries, and Euro-GASP members.

In 2022, 23 Euro-GASP countries submitted complete AMR data for 4396 *N. gonorrhoeae* isolates (one isolate per gonorrhoea episode) to TESSy. However, in order to reduce biases caused by countries reporting more gonococcal isolates than requested, in the present study the number of included gonococcal isolates from the over-reporting countries was reduced, while all gonococcal isolates from the other countries were included. Briefly, seven countries reported data for more *N. gonorrhoeae* isolates than requested (>200 isolates), and only the first 200 *N. gonorrhoeae* isolates collected from 1st September, 2022 in these country were included in the analysis of the present paper. The number of *N. gonorrhoeae* isolates included in the present study (n = 3008), divided by country is shown in Table 1.

**Table 1:** Number of *Neisseria gonorrhoeae* isolates reported to the European Surveillance System (TESSy) in 2022, *N. gonorrhoeae* isolates included in the present Euro-GASP 2022 study, and *N. gonorrhoeae* isolates requested, by country.

Country^a^	Number of isolates reported to TESSy	Number of isolates in present study (%)	Number of isolates requested in present study^b^
Austria	377	200 (53.1)	200
Belgium	200	200 (100)	200
Bulgaria	12	12 (100)	100
Czech Republic	111	111 (100)	200
Denmark	135	135 (100)	200
Estonia	8	8 (100)	100
Finland	91	91 (100)	100
France	220	200 (90.9)	200
Germany	200	200 (100)	200
Greece	100	100 (100)	100
Hungary	122	122 (100)	200
Iceland	63	63 (100)	100
Ireland	294	200 (68.0)	200
Italy	100	100 (100)	200
Malta	61	61 (100)	100
The Netherlands	572	200 (35.0)	200
Norway	827	200 (24.2)	200
Poland	15	15 (100)	100
Portugal	110	110 (100)	200
Slovakia	80	80 (100)	100
Slovenia	285	200 (70.2)	200
Spain	213	200 (93.9)	200
Sweden	200	200 (100)	200
**Total**	**4396**	**3008 (68.4)**	**3800**

Euro-GASP, European Gonococcal Antimicrobial Surveillance Programme.

a All countries except Bulgaria and Finland were included in the Euro-GASP 2016 study,^8^^,^^9^ which also included low numbers of isolates from Croatia, Cyprus, Luxembourg and the UK, and all countries except Bulgaria in the Euro-GASP 2019 study,^1^ which also included low numbers of isolates from Croatia, Latvia and the UK.

b Euro-GASP countries are requested to report data on 100 gonococcal isolates each year if 100 isolates represent ≥10% of the total number of reported gonorrhoea cases in a year, and 200 isolates if 100 isolates represent <10% of the total number of reported gonorrhoea cases in a year.

### Antimicrobial susceptibility testing

Antimicrobial susceptibility testing was performed using MIC gradient strip tests (mainly Etest) to determine the minimal inhibitory concentration (MIC; mg/L) for ceftriaxone, cefixime, azithromycin, and ciprofloxacin. The MIC results were interpreted using clinical resistance breakpoints from the European Committee on Antimicrobial Susceptibility Testing (EUCAST): cefixime/ceftriaxone resistance, MIC >0.125 mg/L and ciprofloxacin resistance, MIC >0.06 mg/L. For azithromycin, the epidemiological cut-off value (ECOFF; MIC >1 mg/L) was used to indicate azithromycin resistance (https://www.eucast.org/clinical_breakpoints, v15.0).

### Statistical analysis

Statistical analysis was performed using Stata v18.0. The Z-test was used to determine the significance of the difference between the collected epidemiological data in 2022 versus 2019^1^ and 2016,^8^^,^^9^ and the difference in the proportion of antimicrobial-resistant isolates in 2022 versus 2019^1^ and 2016.^8^^,^^9^ The Mann–Whitney test was used to determine whether the differences in age distribution were statistically significant. The risk factors associated with AMR isolates were assessed using univariate and multivariable logistic regression analyses of odds ratios (ORs). Categorical patient variables used to identify risk groups were as follows: age-group (<25 and ≥25 years), previous gonorrhoea (yes and no), sexual orientation and sex (men-who-have-sex-with-men (MSM), male heterosexuals and females), HIV status (positive and negative) and anatomical site of infection (urogenital, oropharyngeal, anorectal and other). For sufficient cell counts (n ≥ 5), ORs and 95% confidence intervals (CI) were calculated and Pearson's χ2 test was used to measure if these ORs differed significantly from 1. For small cell counts (n < 5), Fisher’s exact test was instead performed. All patient variables, other than anatomical site of infection, were included in the multivariable logistic regression model. Anatomical site of infection was not included as this can be a clinic dependent variable, for example different clinics and different countries can have different strategies/guidelines for which anatomical sites in each patient that should be sampled. Observations missing data for the variables included in the univariate or multivariable model were excluded from the analysis. Statistical significance for all tests was assumed when p < 0.05.

### Ethical approval

All gonococcal isolates were cultured and preserved as part of the countries’ routine diagnostics and/or surveillance (standard care), and isolates and data were submitted to the ECDC through the Euro-GASP surveillance programme with no patient identification information. Accordingly, no separate ethical approval or consent of the patients were required.

### Role of the funding source

The funder had no role in study design, data collection, analysis, interpretation, or report writing.

## Results

Including a maximum of 200 *N. gonorrhoeae* isolates per country, 3008 isolates collected in 2022 from 3008 episodes of gonorrhoea in 23 EU/EEA countries (Table 1) were compared to the latest published Euro-GASP data from 2019 (n = 3239, 26 EU/EEA countries) and 2016 (n = 2559, 25 EU/EEA countries) (Table 2). The majority of specimens in 2022 were collected from male patients (80.8%, 2416/2991); 84.7% (2158/2549) in 2016 and 84.2% (2676/3178) in 2019. Accordingly, there was a decrease in the number and proportion of male specimens and an increase in the number and proportion of female specimens in 2022 compared to both 2019 (p = 0.0004) and 2016 (p = 0.0002). The decrease in 2022 in male specimens was both among heterosexual males (2019, p = 0.054; 2016, p = 0.0002) and MSM (2019, p = 0.019) (Table 2). In 2022, the age of the patients ranged from <1 year (three cases of eye infection) to 75 years, median (MD) age of 30 years (IQR 24–39). Overall, 27.8% (830/3008) of patients were under 25 years (Table 2), and females (MD 25 years, IQR 21–38) were younger than males (MD 31 years, IQR 25–39) (p < 0.0001). In 2830 (94.1%) of the 3008 gonorrhoea episodes, information about specimen type was reported. Briefly, urogenital samples predominated (75.1%, 2126/2830), followed by anorectal samples (13.2%, 373/2830) and oropharyngeal samples (10.1%, 286/2830). Compared to 2019, the proportion of urogenital samples had increased from 71.5% (2076/2904; p = 0.0019), while the proportion of anorectal samples had decreased, 16.4% (475/2904; p = 0.0007) and the proportion of oropharyngeal samples remained at a similar level, 9.0% (262/2904; p = 0.16). The decrease in anorectal specimens was likely a consequence of the decrease in proportion of specimens from MSM in 2022, which is mentioned above (Table 2).

**Table 2:** Gonorrhoea patient characteristics in Euro-GASP, 2022 compared to 2019^1^ and 2016.^8^^,^^9^

	2016^8^^,^^9^	2019^1^	2022	p-value^a^
	No. (%)^b^	No. (%)	No. (%)	2022 versus 2016/2022 versus 2019
**Number of isolates**^c^	2559	3239	3008	
**Sex**				
Male	2158 (84.7)	2676 (84.2)	2416 (80.8)	**0.0002**/**0.0004**
Female	391 (15.3)	502 (15.8)	575 (19.2)	**0.0002**/**0.0004**
Not reported	10	61	17	
**Age (years)**				
<25	668 (26.5)	883 (28.4)	830 (27.8)	0.28/0.58
≥25	1854 (73.5)	2223 (71.6)	2156 (72.2)	0.28/0.58
Not reported	37	133	22	
**Sex and sexual orientation**				
Females	391 (24.4)	501 (25.6)^d^	575 (32.3)	**<0.0001**/**<0.0001**
Heterosexual males	539 (33.6)	545 (27.9)	446 (25.1)	**<0.0001**/0.050
Men who have sex with men	672 (41.9)	908 (46.5)	759 (42.6)	0.68/**0.019**
Not reported	957	1285	1228	
**Site of infection**				
Urogenital	1745 (75.1)	2076 (71.5)	2126 (75.1)	0.98/**0.0019**
Oropharyngeal	153 (6.6)	262 (9.0)	286 (10.1)	**<0.0001**/0.16
Anorectal	329 (14.2)	475 (16.4)	373 (13.2)	0.31/**0.0007**
Other	97 (4.2)	91 (3.1)	45 (1.6)	**<0.0001**/**0.0001**
Not reported	235	335	178	
**Previous gonorrhoea**				
Yes	163 (16.7)	228 (23.2)	196 (26.2)	**<0.0001**/0.16
No	814 (83.3)	753 (76.8)	552 (73.8)	**<0.0001**/0.16
Not reported	1582	2258	2260	
**HIV status**				
Positive	143 (15.8)	139 (13.1)	87 (9.1)	**<0.0001**/**0.0046**
Negative	760 (84.2)	926 (86.9)	871 (90.9)	**<0.0001**/**0.0046**
Not reported	1656	2174	2050	

a Significant differences by Z-test (p < 0.05) are shown in bold letters.

b The percent is based on the reported number of the variable.

c Includes a maximum of 200 isolates per country.

d One female did not report her sexual orientation.

**Fig. 1:**
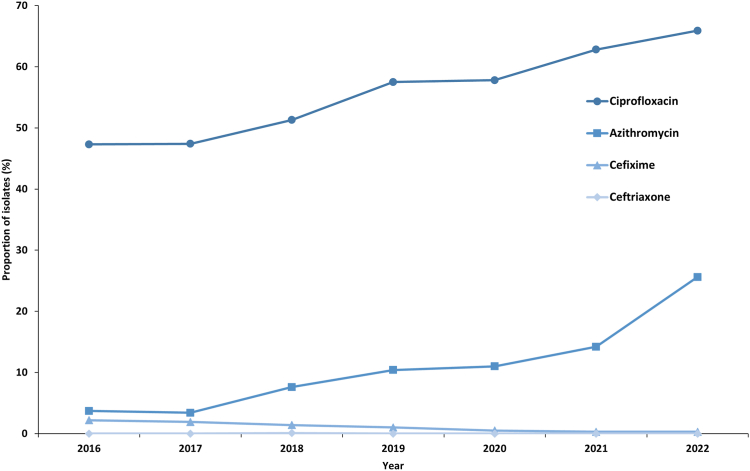
**Proportion (%) of resistant *Neisseria gonorrhoeae* isolates by antimicrobial and year within European Union (including UK until 2019) and European Economic Area, from 2016 to 2022.** All collected Euro-GASP isolates from each year^4^ are included in this graph.

**Fig. 2:**
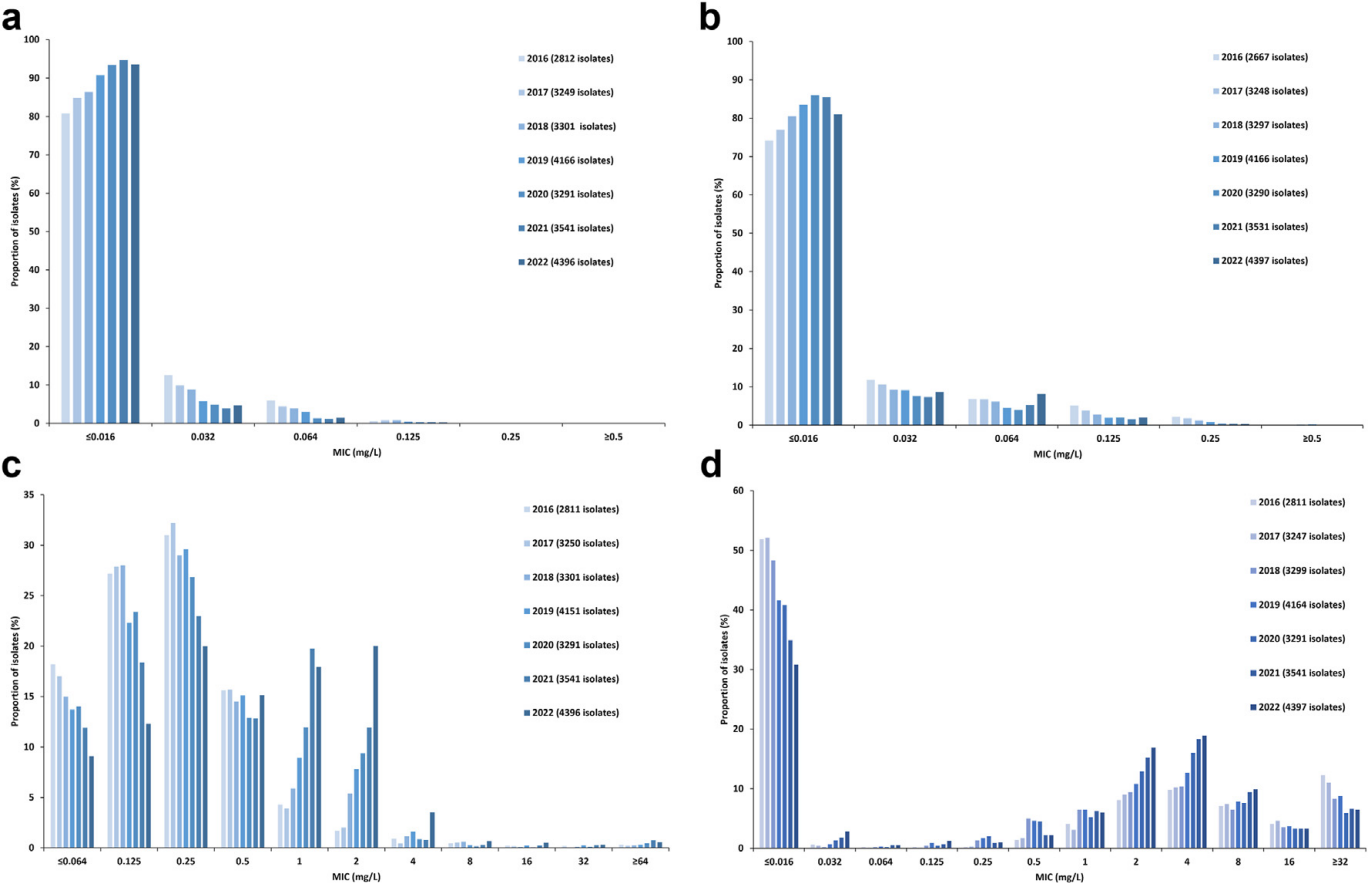
**Minimum inhibitory concentration (MIC; mg/L) distributions for *Neisseria gonorrhoeae* isolates collected within European Union (including UK until 2019) and European Economic Area, from 2016 to 2022.** a) ceftriaxone, b) cefixime, c) azithromycin, and d) ciprofloxacin. All collected Euro-GASP isolates from each year^4^ are included in this graph.

Including all collected Euro-GASP isolates from each year,^4^ the overall resistance to ceftriaxone, cefixime, azithromycin and ciprofloxacin within the EU/EEA (including the UK until 2019) over time and the MIC distributions for these antimicrobials are summarised in Figs. 1 and 2, respectively.

In 2022, ceftriaxone resistance was detected in one isolate (MIC = 0.25 mg/L; 1/3008 (0.03%)) cultured in Germany. This isolate was also resistant to cefixime (MIC = 1 mg/L) and ciprofloxacin (MIC = 4 mg/L), but susceptible to azithromycin (MIC = 0.032 mg/L). Additional epidemiological data was however missing. A single ceftriaxone-resistant isolate in 2022 was comparable with previous years when one urogenital ceftriaxone-resistant isolate was detected each in 2021 (Spain) and 2020 (Belgium) and two urogenital isolates (Belgium and Portugal) in 2019. Also the ceftriaxone MIC distribution has been relatively stable over the past three years (Fig. 2a).

Resistance to cefixime was detected in ten isolates (0.3%, 10/3008) collected in six countries (Sweden (n = 4), Hungary (n = 2), and one isolate each in Austria, Belgium, Germany, and Iceland) in 2022. The proportion of cefixime-resistant isolates (MIC >0.125 mg/L) had decreased from 0.8% (26/3239) in 2019 to 0.3% (10/3008) in 2022 (p = 0.014). Of the ten isolates with cefixime resistance in 2022, only one isolate was also resistant to azithromycin (MIC = 2 mg/L). In 2019, cefixime resistance was higher in females at 1.6% (8/502) compared with 0.7% (4/545) in male heterosexuals and 0.3% (3/908) in MSM (p = 0.045). In 2022, no significant cefixime resistance associations were identified although the proportion of cefixime resistance in females (0.7%, 4/575) remained higher compared to male heterosexuals (0.4%, 4/446) and MSM (0.1%, 1/759), see Table 3. The MIC distribution for cefixime was similar to ceftriaxone with an annual increase in the proportion of highly cefixime-susceptible isolates (MIC ≤0.016 mg/L) from 2016 to 2020. In 2021, this increase in susceptibility stalled and in 2022 (81.7%, 2457/3008) a decrease was observed (Fig. 2b).

**Table 3:** Univariate association of cefixime, azithromycin and ciprofloxacin resistance/susceptibility and patient characteristics in Euro-GASP, 2022.

	Cefixime resistance				Azithromycin resistance				Ciprofloxacin resistance			
	No. (%, 95% CI)	Odds ratio	95% CI	p-value	No. (%, 95% CI)	Odds ratio	95% CI	p-value	No. (%, 95% CI)	Odds ratio	95% CI	p-value
**Age (n = 2986)**												
<25 years (830)	3 (0.4, 0.12–1.06)	1		0.71	198 (23.9, 21.1–26.9)	1			478 (57.6, 54.2–60.9)	1		
≥25 years (2156)	6 (0.3, 0.13–0.61)	0.77	0.19–3.08		548 (25.4, 23.6–27.3)	1.09	0.90–1.31	0.37	1488 (69.0, 67.0–70.9)	1.64	1.39–1.94	**<0.0001**
**Site of infection (n = 2830)**												
Urogenital (2126)	6 (0.3, 0.13–0.61)			0.14^a^	494 (23.3, 21.5–25.1)	1			1381 (65.0, 62.9–67.0)	1		
Anorectal (373)	0 (0.0, 0.0–1.02)				110 (29.5, 25.1–34.3)	1.38	1.08–1.76	**0.0094**	273 (73.2, 68.5–77.4)	1.47	1.15–1.88	**0.0019**
Pharyngeal (286)	3 (1.1, 0.36–3.04)				96 (33.6, 28.3–39.2)	1.67	1.28–2.18	**<0.0001**	191 (66.8, 61.1–72.0)	1.08	0.83–1.41	0.54
Other (45)	0 (0.0, 0.0–7.87)				11 (24.4, 14.2–38.7)	1.07	0.54–2.12	0.85	34 (75.6, 61.3–85.8)	1.67	0.84–3.31	0.14
**Mode of transmission and sex (n =1780)**												
MSM (759)	1 (0.1, 0.02–0.74)			0.24^a^	275 (36.2, 32.9–39.7)	3.88	2.80–5.37	**<0.0001**	546 (71.9, 68.6–75.0)	2.48	1.97–3.14	**<0.0001**
Male heterosexual (446)	2 (0.4, 0.12–1.62)				57 (12.8, 10.0–16.2)	1			293 (65.7, 61.2–70.0)	1.86	1.43–2.40	**<0.0001**
Female (575)	4 (0.7, 0.27–1.77)				115 (20.0, 16.9–23.5)	1.71	1.21–2.41	**0.0022**	292 (50.8, 46.7–54.9)	1		
**Previous gonorrhoea (n = 748)**												
Yes (196)	0 (0.0, 0.0–1.92)			0.74^a^	55 (28.1, 22.2–34.7)	1.34	0.93–1.95	0.12	143 (73.0 66.3–78.7)	0.95	0.66–1.38	0.79
No (552)	1 (0.2, 0.03–1.02)				124 (22.5, 19.2–26.2)	1			408 (73.9, 70.1–77.4)	1		
**HIV status (n =958)**												
Positive (87)	0 (0.0, 0.0–0.44)			b	23 (26.4, 18.3–36.6)	1.21	0.73–2.01	0.45	64 (73.6, 63.4–81.7)	1.1	0.65–1.77	0.77
Negative (871)	0 (0.0, 0.0–0.44)				199 (22.9, 20.2–25.8)	1			628 (72.1, 69.0–75.0)	1		

No, number; CI, confidence interval; MSM, men who have sex with men. Bold numbers indicate significance.

a Fisher’s exact test.

b Not possible to test as both variables are zero.

The percentage of isolates with azithromycin resistance increased from 9.0% (284/3159) in 2019 to 24.9% (749/3008) in 2022 (p = 0.0002). In 2022, azithromycin-resistant isolates were cultured in all 23 participating Euro-GASP countries. Nevertheless, the majority of the azithromycin-resistant isolates had a low-level resistance, i.e., 76.2% (571/749) had an MIC of 2 mg/L and 15.5% (116/749) an MIC of 4 mg/L. The proportion of isolates with high-level azithromycin resistance (MICs of ≥256 mg/L), 0.3% (9/3008), was similar as in 2019 (0.3%, 10/3159). These high-level azithromycin-resistant isolates were cultured in Sweden (n = 3), Finland (n = 2), Norway (n = 2), and one isolate each in Austria and Greece, with eight of the isolates collected in males and one in a female. The countries with the highest proportion of azithromycin-resistant isolates in 2022 were Portugal (59.1%, 65/110), Ireland (47.0%, 94/200), Belgium (43.0%, 86/200), Sweden (43.0%, 86/200), and Poland (40.0%, 6/15). Univariate analyses showed associations between azithromycin resistance and oropharyngeal infection (OR 1.67, CI 1.28–2.18, p < 0.0001) and anorectal infection (OR 1.38, CI 1.08–1.76, p < 0.0094). Additionally, MSM (OR 3.88, CI 2.80–5.37, p < 0.0001) and females (OR 1.71, CI 1.21–2.41, p = 0.0022) were associated with azithromycin resistance, when compared to heterosexual males (Table 3). In the multivariable logistic regression model, only azithromycin resistance remained associated with MSM (OR 2.85, CI 1.33–4.73, p = 0.0040). The proportion of azithromycin susceptible isolates with MICs of ≤0.064, 0.125 and 0.25 mg/L decreased since 2019 with an increase in the proportion of isolates with azithromycin MICs of 1, 2 and 4 mg/L (Fig. 2c).

Ciprofloxacin resistance levels have steadily increased over the years, in 2022 65.8% (1980/3008) of the isolates were resistant compared to 57.4% (1665/2884) in 2019, p = 0.0002 (Fig. 1, Fig. 2d).

## Discussion

The antimicrobial susceptibility and resistance trends for ceftriaxone, cefixime, azithromycin, and ciprofloxacin have remained relatively consistent in Euro-GASP during the recent years. Encouragingly, the susceptibility to the extended-spectrum cephalosporins (ESCs), ceftriaxone and cefixime, remains high. However, in 2022, the proportion of highly ESC-susceptible isolates decreased while the proportion of isolates with decreased ESC susceptibility increased, which was in contrast to the trend observed since 2016.^1^^,^^8^^,^^9^ These changes warrant close monitoring in the coming years. While ceftriaxone resistance thus far has been rare in Europe, sporadic cases and treatment failures have been reported in numerous European countries and globally in recent years,^10^ and six ceftriaxone-resistant gonorrhoea cases (from the UK (n = 3), Norway (n = 2), and Belgium (n = 1)) were reported to the ECDC EpiPulse platform^11^ in 2023. Furthermore, in some countries such as Vietnam,^12^ Cambodia^13^ and China^14^ the ceftriaxone-resistant and multidrug-resistant FC428 clone^10^^,^^15^ as well as other ceftriaxone-resistant strains are now in sustained transmission. In these countries, the level of ceftriaxone resistance is high, and the number of reports of ceftriaxone-resistant strains imported to Europe from these countries have increased in recent years.^10^, ^11^, ^12^, ^13^, ^14^, ^15^, ^16^, ^17^, ^18^, ^19^, ^20^, ^21^, ^22^ Novel antimicrobials for treatment of gonorrhoea are clearly required and, most recently, the oral drugs zoliflodacin^23^ and gepotidacin^24^ showed non-inferiority in their phase 3 randomized controlled clinical trials compared to ceftriaxone and azithromycin dual therapy in the treatment of urogenital gonorrhoea.

In 2022, the most dramatic change in Euro-GASP was the increase in azithromycin resistance, i.e., from 9% in 2019^1^ to 25% in 2022, with azithromycin-resistant isolates collected across all participating countries. Nevertheless, the majority of azithromycin-resistant isolates were displaying low-level resistance, 76% of the azithromycin-resistant isolates exhibited an MIC of 2 mg/L. It remains unknown if gonococcal infections caused by isolates with low-level resistance can be cleared with a single oral dose of azithromycin 2 g. Promisingly, it has been recently demonstrated using a dynamic *in vitro* hollow fibre infection model that *N. gonorrhoeae* isolates with low-level azithromycin resistance (MIC = 2 mg/L) can be effectively treated with azithromycin 2 g single oral dose.^25^ Nevertheless, no azithromycin monotherapy regimen is recommended in the European gonorrhoea management guideline.^7^ Notably, using azithromycin monotherapy isolates with relatively low azithromycin MICs have caused treatment failure and azithromycin resistance can be selected during treatment.^26^^,^^27^ Regarding high-level azithromycin resistance (MICs of >256 mg/L), it is encouraging that the number of high-level resistant isolates in 2022 remained constant compared to previous years. The global trend, along with the Euro-GASPs findings, is however increasingly concerning due to the rapid increase in azithromycin resistance over the past 5–10 years.^1^^,^^3^^,^^4^^,^^6^^,^^22^ This includes also sustained national and international transmission of high-level azithromycin-resistant (MIC ≥256 mg/L) *N. gonorrhoeae* strains with concomitant resistance to ceftriaxone.^13^^,^^16^^,^^17^^,^^19^

Alongside with increasing resistance to azithromycin and ciprofloxacin the most prominent trend seen in 2022 was the increase of *N. gonorrhoeae* isolates cultured from young females. The majority of samples in 2022 were collected from male patients, as it has been in all previous Euro-GASP collections.^4^ However, in 2022 a significant increase in number and proportion of isolates from females was observed. This was also reflected in an increase in urogenital samples and decrease in anorectal samples. The females with their median age of 25 years were also younger than the males with median age of 31 years. These findings correspond well to the increase in reported overall gonorrhoea cases in 2022 in the EU/EEA and especially among younger heterosexual females in the second half of 2022 and continuing in early 2023 that was recently reported from several European countries through the EpiPulse and Early Warning and Response platforms coordinated by ECDC.^11^^,^^28^^,^^29^ Nerlander et al.^29^ recently described the trends in gonorrhoea notifications in the EU/EEA since 2015 with sharp increases in reported cases among women aged 20–24 years, accompanied with increases in heterosexual men aged 20–24 and 25–59 years. Whereas the number of reported cases among MSM has been gradually increasing since 2015 across all age groups, except those aged 15–19 years. Focusing on women aged 20–24 years, the country-specific gonorrhoea notifications in Denmark, Iceland, Ireland, the Netherlands, Norway, Portugal, Slovenia and Spain dramatically increased in either the second half of 2022 or the first half of 2023, while Finland, Italy and Sweden saw less dramatic increases and Estonia, Hungary and Latvia did not see any increases in gonorrhoea notifications.^29^

The limitations of the present study, and accordingly Euro-GASP, include the limited number and suboptimal representativeness of *N. gonorrhoeae* isolates available in some EU/EEA countries, e.g., three countries submitted less than 20 isolates each. Nevertheless, 23 EU/EEA countries and *N. gonorrhoeae* isolates from more than 4% of the 70,881 confirmed gonorrhoea cases in EU/EEA in 2022 were included in the present study. Continuous work to have the remaining seven EU/EEA countries (Cyprus, Croatia, Latvia, Liechtenstein, Lithuania, Luxemburg and Romania) included in Euro-GASP from 2023 and beyond, and to increase the overall coverage of isolates in all EU/EEA countries are in progress. A main factor resulting in a limited number of *N. gonorrhoeae* isolates in many EU/EEA countries is the widespread use of molecular diagnostics for gonorrhoea, which has caused a lack of *N. gonorrhoeae* culture in several EU/EEA countries. Another limitation is the suboptimal coverage and completeness of reporting the epidemiological and clinical variables, thereby compromising the precision of certain estimates, especially for specific sub-groups with small sample sizes. Continuous work to improve this suboptimal coverage and completeness of the reporting is performed by Euro-GASP and ECDC in general. Accordingly, the suboptimal reporting is strongly emphasized and discussed in annual Euro-GASP meetings, European STI network meetings, Euro-GASP laboratory trainings, and during country visits. The ‘Response plan to control and manage the threat of multi- and extensively drug-resistant gonorrhoea in Europe. 2019 update’^30^ also stresses the importance of appropriate reporting of epidemiological and clinical variables and the indicators for implementation of the response plan, including appropriate collection of patient data, are also monitored. Ultimately, a proactive national sentinel clinic surveillance may support more systematic data collection in some countries. A final limitation is the predominance of urogenital site-derived isolates in Euro-GASP and the limited number of isolates obtained from anorectal and oropharyngeal sites that might contribute to the study's constraints. Despite the mentioned limitations, a previous representativeness analysis demonstrated that Euro-GASP adequately reflects the AMR situation for *N. gonorrhoeae* in the EU/EEA^31^ as well as the epidemiological trends for gonorrhoea, as indicated in a recent publication by Nerlander et al.^29^

In conclusion, the susceptibility trends observed for the antimicrobials tested within Euro-GASP, including ceftriaxone, cefixime, azithromycin, and ciprofloxacin, have remained relatively consistent over recent years. Notably, the susceptibility to the ESCs, ceftriaxone and cefixime, has remained high, although the MIC values for both these ESCs increased in 2022 and the number of occasional reports of ceftriaxone-resistant and cefixime-resistant isolates in EU/EEA countries^11^^,^^16^^,^^17^^,^^19^^,^^21^ appear to be increasing, which warrant further monitoring. Of particular concern is the rapidly increasing azithromycin resistance observed in 2022, which emphasises the importance of a review of the treatments recommended in the 2020 European gonorrhoea management guideline.^7^ Finally, another significant trend observed in 2022 was the increase in gonorrhoea samples collected from young females, reflecting an overall increase in gonorrhoea cases in this demographic. This trend is corroborated by reports from several European countries^29^ and underscores the need for continued surveillance and response efforts. Notably, a Euro-GASP 2023 WGS study has been initiated. This WGS study will address both the fluctuations in gonococcal AMR, linked to epidemiological variables of the gonorrhoea patients, and the changing gonorrhoea epidemiology in Europe. Nevertheless, further work to improve the suboptimal coverage and completeness of the reporting of several key epidemiological variables, including sexual orientation, in Euro-GASP is imperative for appropriate and detailed analysis. Finally, new antimicrobials for the treatment of gonorrhoea are urgently needed and zoliflodacin and gepotidacin recently showed non-inferiority compared to a ceftriaxone plus azithromycin regimen for the treatment of uncomplicated gonorrhoea in phase 3 randomised controlled clinical trials. However, the only long-term solution for effective management and control of gonorrhoea is likely a gonococcal vaccine.

## Contributors

SJ, MJC, MJR, CK, and MU designed, initiated, and coordinated the study. The Euro-GASP study group members collected gonococcal isolates and patient data, and read and commented on the manuscript. SJ, DS, and MD performed the main laboratory work. The first author (SJ) and corresponding author (MU) verified all the data in the study. SJ analysed and interpreted the data, and wrote a first draft of the paper with support by MU. All authors (SJ, MJC, DS, MJR, MD, CK, MU) had full access to the data, read, commented on and approved the final manuscript, and were responsible for the decision to submit for publication.

## Data sharing statement

Most data collected and analysed in this study are included in the main paper. However, remaining datasets can be made available from the corresponding author after publication on reasonable request.

## Declaration of interests

European Centre for Disease Prevention and Control (not-for-profit EU agency) has a Framework Service Contract with Örebro University Hospital, Sweden to coordinate Euro-GASP. UK Health Security Agency is subcontracted in this work. All authors declare that they have no conflicts of interests.
